# Attachment of Tridentate Ligand onto the Polyhedral Oligomeric Silsesquioxanes as an Efficient Strategy to Capture La(III) Ions: A Comparative Study of Homogeneous and Heterogeneous Systems Using Potentiometric Titration

**DOI:** 10.1021/acsomega.5c06716

**Published:** 2025-10-21

**Authors:** Débora de Freitas Brotto, Iago de Souza Reis, Adolfo Horn, Bruno Szpoganicz

**Affiliations:** † Department of Chemistry, 28117 Universidade Federal de Santa Catarina (UFSC), Avenue Desembargador Vitor Lima (s/n), 88040-900 Florianópolis, Santa Caterina, Brazil

## Abstract

Developing sustainable methods for lanthanide ion recovery is a relevant area of research that seeks to promote the efficient reuse of these valuable chemical elements present in several electronic devices. Adsorption techniques using materials functionalized with chelating groups, such as modified Polyhedral Oligomeric Silsesquioxanes (POSS) matrices, may play a crucial role in capturing these metals. This work investigates the coordination of the lanthanum­(III) ion with two tridentate organic ligands: bis­(pyridin-2-ylmethyl)­amine (L1) and (pyridin-2-ylmethyl)­(thiophen-2-ylmethyl)­amine (L2). The results revealed stronger interactions for L1, with higher formation constants (9.0 for [LaL1]^3+^ and 16.9 for [La­(L1)_2_]^3+^) compared to L2 (4.37 for [LaL2]^3+^). Further studies on ligand L1 attached to silsesquioxane (POSS-L1) showed significant metal ion interactions, showing the formation of [LaPOSS-L1]^3+^ (81.0%) at pH 5.42 and La­(OH)_3_POSS-L1 above pH 8.0, with a formation constant of 10.33, which is higher than that for free L1. These results demonstrate the efficient binding of La­(III) to the POSS-L1 in a heterogeneous system. The modified silsesquioxane matrix presents a promising alternative for lanthanide ion adsorption, contributing to sustainable metal recovery strategies.

## Graphical abstract

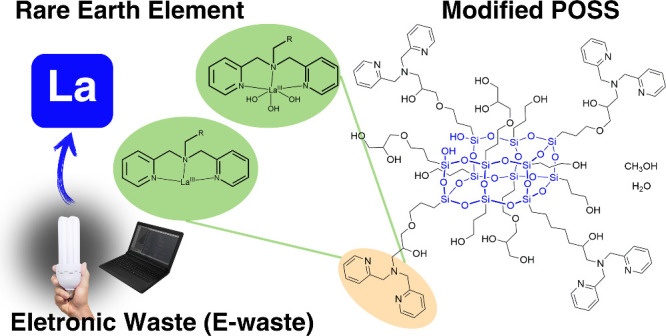

## Introduction

Lanthanum is the third most abundant rare-earth element (REE), known for its fluorescent properties and magnetic potential.^1^ It is used in various fields, including steel production, lubrication, batteries, electrocatalysis, computers, solar cells, wind turbines, lasers, optical glasses, LEDs, and fluorescent lamps.^1–11^ For example, among the REEs present in fluorescent lamps, lanthanum is one of the most abundant, accounting for 34% (wt). Other REEs are cerium, terbium, yttrium, and europium.^12^

Various methodologies for the recovery of rare earth elements from lamps, electric and electronic equipment have been discussed in the literature, including solvometallurgical/hydrometallurgical leaching with acid, pyrometallurgy, solvent extraction, alkaline fusion with acid leaching, calcination, electrochemical, and biometallurgy.^3,13–18^ These methods produce waste that requires proper treatment and incurs high costs due to the volume of reagents and equipment needed for their implementation. Therefore, developing new, effective, and low-cost methods for recovering lanthanide ions from solutions is a strategic approach for reusing rare earth elements (REEs) that would otherwise be discarded. In this context, adsorption has been chosen as a suitable process for recovering REEs from an environmental perspective.^19,20^

Selecting the ideal conditions for metal adsorption is a critical process. Key variables that need careful adjustment include the optimal pH values, the interactions and distribution of species in solution, and the choice of adsorbent, particularly the species that has the highest affinity for the metal ion. For this proposal, potentiometric titration studies are vital, as they help determine the best experimental parameters for adsorption studies.^20,21^

Silsesquioxanes (SSQs) are a class of silicon–oxygen compounds with the general empirical formula (RSiO_1.5_)_*n*_, where R is an organic substituent such as alkyl or aryl groups. These compounds are characterized by a cage-like or ladder-like three-dimensional framework composed of silicon and oxygen atoms, forming a siloxane (Si–O–Si) network with pendant organic groups attached to silicon atoms.^22^ One of the most studied subclasses is polyhedral oligomeric silsesquioxanes (POSS), which have well-defined, nanometer-sized cage structures and are used as nanoscale building blocks in hybrid organic–inorganic materials.^23,24^ POSS exhibits remarkable characteristics that include enhanced mechanical, thermal, and surface properties, as well as low dielectric constants.^25^ They are used in polymer synthesis,^26^ composites,^27^ catalysis,^28^ electronic optical materials,^29^ electrochemical sensors, and biosensors.^30^ Their structure can be arranged in the following forms: random, ladder-like, and cage-like compounds.^31,32^ Due to their thermal stability, robust structure, and versatile applications, these structures have undergone various modifications, with the general formula (RSiO_1.5_)*_n_*, tailored to their applications.^33^ The use of the POSS matrix functionalized with organic ligands is already known for applications such as fluorescent sensors, liquid crystals, photoresist materials, organic semiconductors, drug/gene delivery systems, biomaterials (bone tissue), emulsifiers, tissue engineering, and wound healing.^25,34,35^

Due to the interesting POSS properties, this study focused on the synthesis of a functionalized POSS containing tridentate ligand and the evaluation of its capability to bind La­(III) ions in comparison with the free ligand. Initially, two tridentate ligands (Figure 1) were synthesized, and their coordination behavior in the presence of La­(III) ions was studied in solution employing potentiometric titration. The ligand that showed the higher formation constant (L1) was employed in the preparation of a POSS (Figure 2), resulting in a solid adsorbent. The equilibrium involving the interaction POSS-L1-La was investigated, allowing for a comparison of the interaction capacity between the homogeneous and heterogeneous systems with the La­(III) ion.

**Figure 1:**
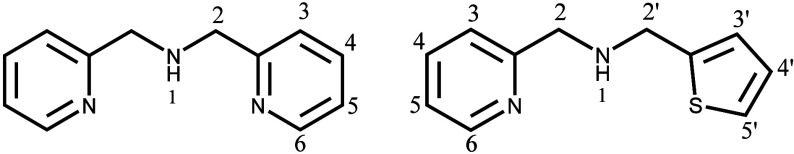
Structures of the ligands: L1 = bis­(pyridin-2-ylmethyl)­amine and L2 = (pyridin-2-ylmethyl)­(thiophen-2-ylmethyl)­amine. The numbers indicate the hydrogen atoms that were observed in the NMR analyses.

**Figure 2:**
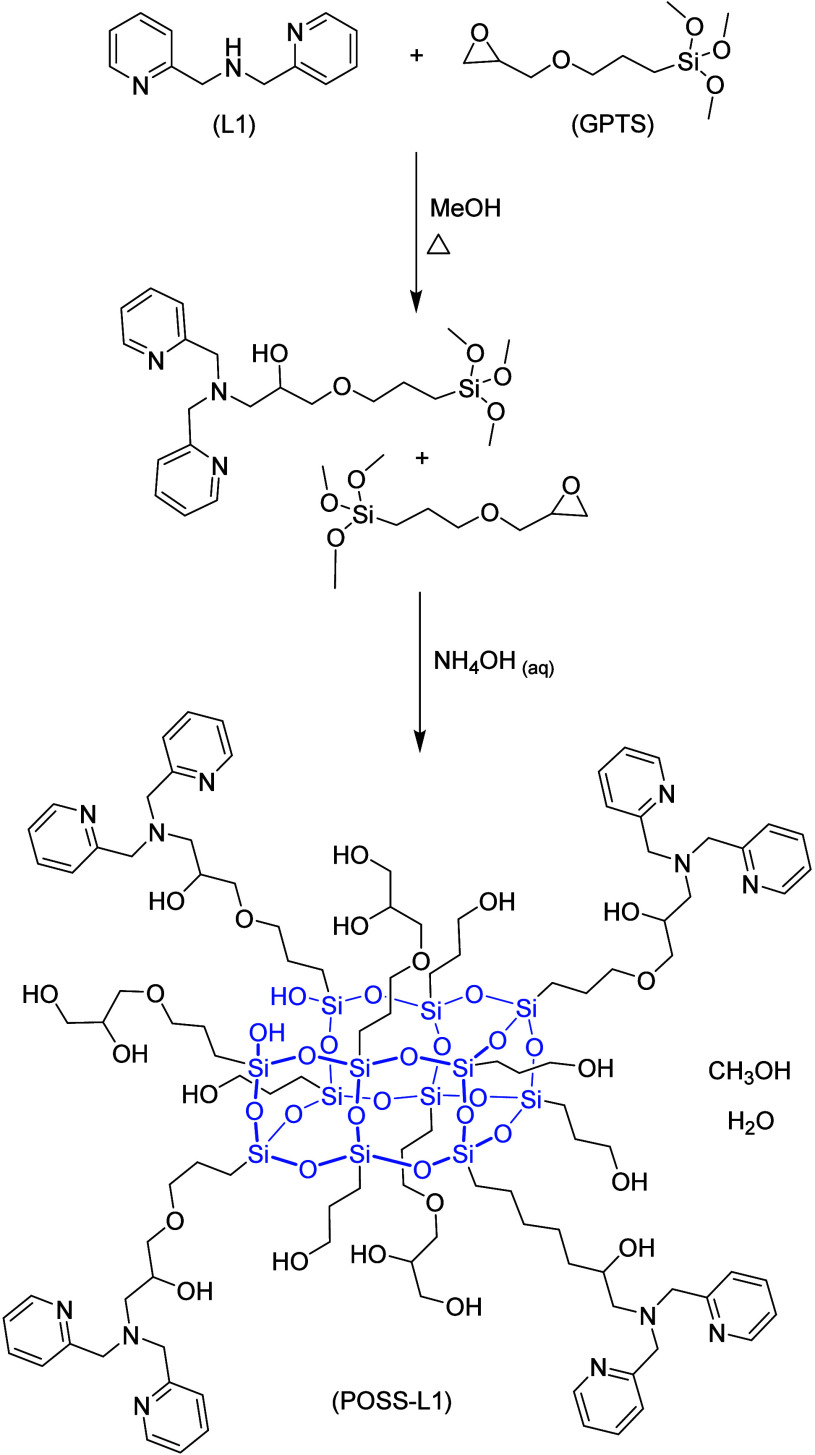
Synthesis reaction of the POSS-L1.

## Materials and Methods

### Methods and Instrumentation

The reagents and solvents employed in the syntheses and characterizations were obtained from commercial sources (Sigma-Aldrich) and were used without prior purification. The ligands and the silsesquioxane matrix were characterized by using infrared spectroscopy (IR), nuclear magnetic resonance (NMR) spectroscopy, CHN elemental analysis, and potentiometric titration techniques. ^1^H NMR spectra were recorded on a Varian FT-NMR spectrometer operating at 400 and 100 MHz.

The ^29^Si and ^13^C CP-MAS NMR experiments were performed on a Bruker Avance III HD 400WB spectrometer operating at 9.4 T using a double resonance probe of 4 mm. The spectra of ^29^Si CP-MAS NMR were recorded using an excitation pulse of 3.4 μs, a contact time of 40 ms, and a relaxation time of 3 s. During data acquisition, all spectra were acquired with a spinal64 pulse sequence proton decoupling. The ^13^C CP-MAS spectra were recorded using a contact time of 2.0 ms and a relaxation time of 3 s. During data acquisition, all spectra were acquired with TPPM proton decoupling. The chemical shifts are reported relative to TMS. The CHN analysis data were obtained by using a PerkinElmer model 2400 Series II CHNS-O elemental analyzer.

The potentiometric titrations were carried out in an automatic Titrino Plus 350 titrator (Metrohm) equipped with a combined glass electrode (Ag/AgCl) in a thermostatic cell maintained at 25 ± 0.1 °C, under continuous stirring and an inert argon atmosphere, free of CO_2_ (purified with a 0.1 mol L^–1^ KOH solution) in an ethanol/water mixture (70:30; v/v). Calibration was performed by titration of a dilute 0.01 mol L^–1^ HCl solution. A volume of 20 mL of ethanol/water solution (70:30; v/v), with p*K*_w_ = 14.71,^36^ was used. For the ligand/POSS matrix samples (0.08–0.09 mmol) in their free and metal-bound forms (0.02–0.04 mmol), titrations were performed using CO_2_-free KOH (0.100 mol L^–1^), standardized with potassium biphthalate. The ionic strength was maintained constant at 0.10 mol L^–1^ with KCl.

The lanthanum chloride solution was standardized using Eriochrome T as an indicator and an excess of EDTA (0.010 mol L^–1^), which was heated close to the boiling point. The excess EDTA was titrated with a standard zinc chloride solution (0.010 mol L^–1^) using the same indicator. The pH was maintained at 10.0 with an ammonium chloride/ammonia buffer.^37,38^

The results of the potentiometric titration studies, conducted in triplicate, were refined using the BEST7 program^39,40^ and the species distribution diagrams were obtained using the SPECIES program and plotted in Origin 9.0. The protonation constants (log *K*_*n*_^H^) of the ligands were determined and are defined by eq 1.

$$H_{n-1}L+H^{+}⇌H_{n}L,K=\frac{\left[H_{n}L\right]}{\left[H_{n-1}L\right]\left[H^{+}\right]}$$ 1

Where *n* = 1 and 2, referring to the two protonation steps of each ligand.

In the calculations of the constants involving the complexation with La­(III), all protonation equilibria of the ligands and metal hydrolysis reactions were taken into account.^41^

#### Synthesis

The syntheses of the ligands and the POSS matrix have already been described.^42–45^ The ligands L1 and L2 were characterized by ^1^H and ^13^C NMR. The POSS was characterized by CHN elemental analysis and ^29^Si, ^13^C solid state NMR.

##### Ligand Bis­(pyridin-2-ylmethyl)­amine: (L1)

The synthesis of L1 (Figure 1) was carried out according to the literature.^43^ Yield: 70% (brown oil). ^1^H NMR (CDCl_3_), δ (ppm): 3.23 (s, 1H, N–H); 3.98 (s, 4H, NCH_2_Py); 7.14 (t, 2H, H_5_–Py); 7.35 (dd, 2H, H_3_–Py); 7.62 (td, 2H, H_4_–Py); 8.55 (dd, 2H, H_6_–Py).

##### Ligand (Pyridin-2-ylmethyl)­(thiophen-2-ylmethyl)­amine: (L2)

The ligand L2 (Figure 1) has already been reported in the literature.^44^ The oily material obtained was purified on a silica gel chromatographic column using ethyl acetate as the eluent. Yield: 26.72% (brown oil). ^1^H NMR (CDCl_3_): δ (ppm) = 2.07 (s, 1H, NH), 3.96 (s, 2 H, NHCH_2_th), 4.04 (s, 2 H, NHCH_2_py), 6.95–6.97 (m, 2 H, H_4′_-th and H_3′_-th), 7.16–7.19 (m, 1H, H_5_–py), 7.22 (dd, 1 H, H_5′_-th), 7.32 (d, 1 H, H_3_–Py), 7.62 (td, 1 H, H_4_–Py), 8.55 (dd, 1 H, H_6_–Py).

##### POSS-L1Matrix

The product was obtained through the reaction of ligand L1 (1.2145 g; 6.095 mmol) with (3-glycidyloxypropyl)­trimethoxysilane (GPTS) (2.8810 g; 12.19 mmol) under reflux for 72 h in a methanolic solution (Figure 2). Afterward, the solvent was evaporated under vacuum. To the isolated product, 19.37 mL of 28% NH_4_OH was added, and the mixture was kept under reflux for an additional 48 h, which led to the formation of a solid. The reaction mixture was then concentrated, filtered, washed with methanol, and dried under vacuum, yielding 1.523 g of a light brown powder.^45^ IR: 2935 and 2867 cm^–1^ (νC–H, symmetric and asymmetric stretching vibrations); 1592, 1478, and 1437 cm^–1^ (νC=N and νC=C); 1113 and 765 cm^–1^ (Si–O–Si, asymmetric and symmetric stretching);^46^ ^13^C NMR (solid state): 150.84–125.46 ppm (aromatic C); 76.73–59.27, 25.67, 20.78, and 11.46 ppm (aliphatic C); ^29^Si NMR (solid state): −67.73 (C–Si­(OSi)_3_) and −58.32 ppm (C–Si­(OSi)_2_OH); elemental CHN analysis: calcd/found for C_106_H_178_N_12_O_43_Si_12_ (2645.63 g mol^–1^); C: 48.12/48.06%; H: 6.78/6.73%; N: 6.35/6.28%. Degree of functionalization (mmol of ligand/grams of POSS-L1): 1.50 mmol g^–1^.

All of the metal complexes of lanthanum­(III) ion were generated in solution during the potentiometric titration experiments.

## Results and Discussion

### Synthesis and Characterization of Ligands and POSS-L1

The ^1^H NMR spectrum of L1 (Figure S1) confirms the observability of the proposed molecule and its purity. The hydrogen of the secondary amine is observed as a broad singlet at 3.23 ppm (s, 1H, N–H), while the singlet at 3.98 ppm is related to the py-CH_2_–N hydrogen atom. Additionally, the hydrogens of the pyridinic rings show chemical shifts at 7.14 ppm (t, 2H), 7.35 ppm (dd, 2H), 7.62 ppm (td, 2H), and 8.55 ppm (dd, 2H).^43^

The ^1^H NMR analysis of L2 (Figure S2) shows, as its main feature, signals corresponding to the hydrogens of the thiophene group with characteristic signals at 6.95–6.97 ppm (m, 2H) and 7.22 ppm (dd, 1H). Additionally, signals similar to those of L1 are observed, with slight shifts, corresponding to the hydrogens of the following groups: pyridine at 7.16–7.19 ppm (m, 1H), 7.32 ppm (d, 1H), 7.62 ppm (td, 1H), and 8.55 ppm (dd, 1H, 6′py); amine group hydrogen at 2.07 ppm (s, 1H, NH); aliphatic carbons’ hydrogens: at 3.96 ppm (s, 2H, NHCH_2_th) and 4.04 ppm (s, 2H, NHCH_2_py).^44^

The POSS-L1 was characterized by solid state NMR (^13^C and ^29^Si). In the ^13^C NMR spectrum of the POSS-L1 matrix (Figure 3), the presence of aromatic carbon signals from L1 is observed between 150.84 and 125.46 ppm. The aliphatic carbon atoms observed at 76.73–59.27 range, are related to CH_2_ and CH groups.^45,47,48^ The signals at 25.67, 20.78, and 11.46 ppm are associated with carbon atoms in proximity to the silicon atom.^45,49^ ^29^Si NMR spectrum (Figure 3) displays the characteristic resonances of T^2^ (C–Si-(OSi)_2_OH) and T^3^ (C–Si­(OSi)_3_) silicon sites at chemical shifts of −58.32 and −67.73 ppm, respectively. The dominance of the T^3^ sites corroborates the presence of a highly organized central framework in POSS-L1, sustained by covalent bonding Si–O–Si.^45,49,50^ Such data confirm the presence of incorporation of ligand L1 in the siloxysilane structure. Additionally, the IR analysis (Figure S3) confirms the condensation/polymerization of GPTS into POSS-L1 by the presence of an intense band at 1113 cm^–1^ and 765 cm^–1^, characteristic of the asymmetric and symmetric stretching, respectively, of the siloxane group (Si–O–Si).^45,51^

**Figure 3:**
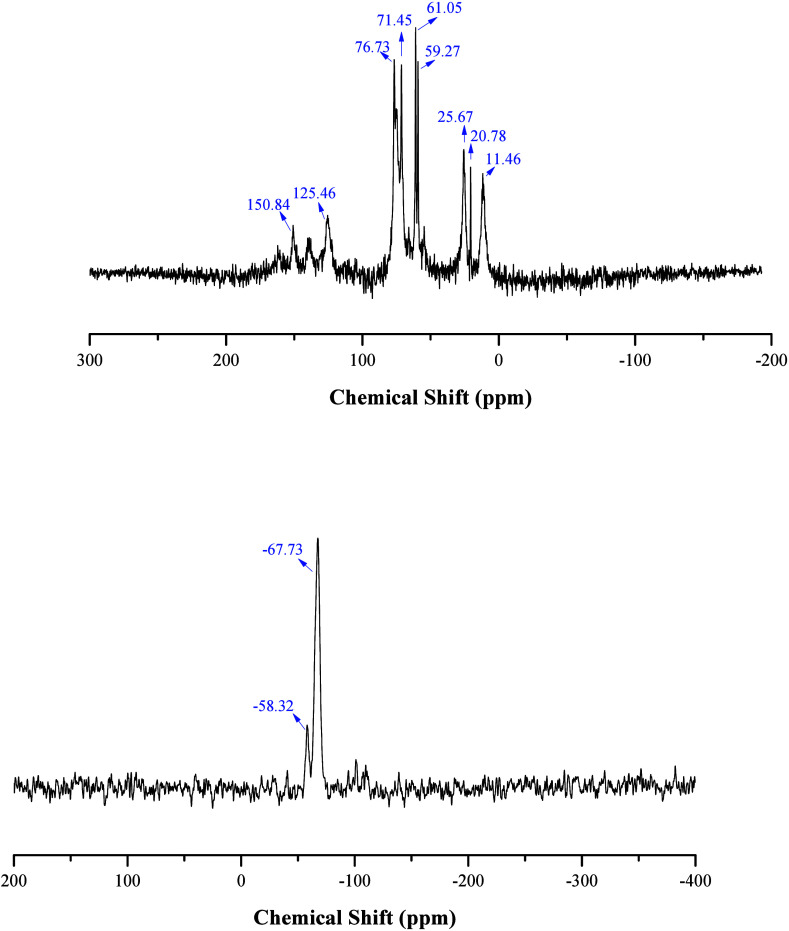
^13^C (top) and ^29^Si solid state NMR spectra of POSS-L1.

### Potentiometric Titration: Equilibrium Studies

Potentiometric titration was employed for obtaining acid dissociation constants and stability constants of the complexes formed in solution.^52^ Most of the titration studies are conducted in an aqueous solution. However, due to the solubility issues of organic ligands and their metal complexes, a mixture of ethanol/water was used. The use of mixture of solvents have been used, such as ethanol,^53,54^ ethanol/water (0–60:100–40; 70:30, 44:56; v/v),^55–58^ acetonitrile/water (50:50; v/v),^59,60^ dimethyl sulfoxide/water (60:40 e 40:60 v/v;),^61^ methanol/water (90:10; v/v),^62^ methanol/toluene (95:5; v/v),^63^ and acetone/water (50:50; v/v).^64^

### Ligands

The potentiometric titration studies were conducted to understand the behavior of both ligands in solution and in the complexation reactions with the lanthanum­(III) ion. The p*K*_a_ values obtained for L1, 6.30 and 1.48, are attributed to the amine and pyridine groups, respectively, and are compared to the literature values in aqueous systems or water/organic solvent mixtures.^65^ The small differences are due to the solvent mixture used, with a decrease in p*K*_a_ observed when up to 80% ethanol is added, as seen in ligands particularly in ligands that contain nitrogenated groups.^65–67^ In the potentiometric curve for L1 (Figure 4a, black line), two buffer regions can be observed, corresponding to the protonation of the amino group and one of the two pyridine groups, as the p*K*_a_ of the other pyridine falls within a more acidic pH range.

**Figure 4:**
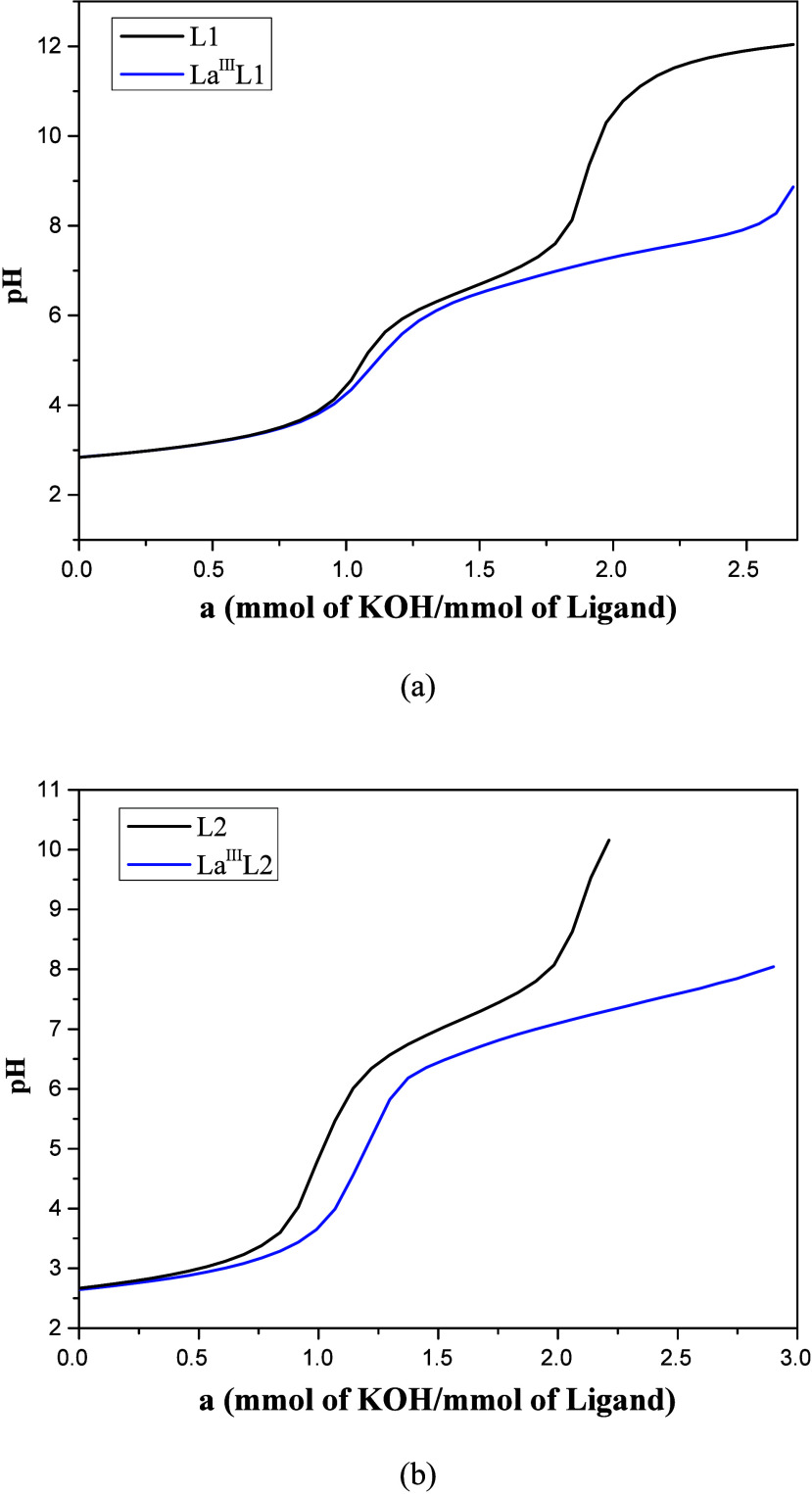
Experimental curves of the potentiometric titrations of ligands (a) L1 and (b) L2 and their complexes with La­(III).

The p*K*_a_ values obtained for L2 were 6.93 and 1.42, attributed to the protonation of the secondary amine and pyridine group, respectively. The p*K*_a_ of the thiophene group was not determined as its protonation occurs in a more acidic region. The curve of L2 (Figure 4b, black line), as a function of mmol of base added per mmol of ligand shows the first and second protonation of the amine and pyridine groups.

### Complexes and POSS-L1

A study was performed to evaluate the differences in the formation constants of complexes formed between La­(III) and ligands L1 and L2 using potentiometric titration. As shown in Figure 4, a decrease in the titration curves of L1 and L2 in the presence of lanthanum­(III) ion (Figure 4) is observed, but it is more significant for the curve of L1 in the presence of the metal ion, indicating a stronger affinity.

The equilibrium reactions of complexation of La­(III) by ligand L1 and the species detected appear in Figure 5, along with their respective formation constant values. The species 1:1 [LaL1]^3+^ and 1:2 [La­(L1)_2_]^3+^ metal/ligand ratio were detected, and the formation constants are shown in Table 1. The formation constants of the [LaL1]^3+^ and [La­(L1)_2_]^3+^ complexes are consistent with those reported by Orvig and collaborators,^68^ who investigated lanthanum­(III) complexes with dipicolinic acid amine-derived ligands, particularly the H_2_dpa ligand (6,6′-(azanediyldimethylene)­dipicolinic acid). These findings further support the interaction between the metal ion and the L1 ligand observed in this study.

**Figure 5:**
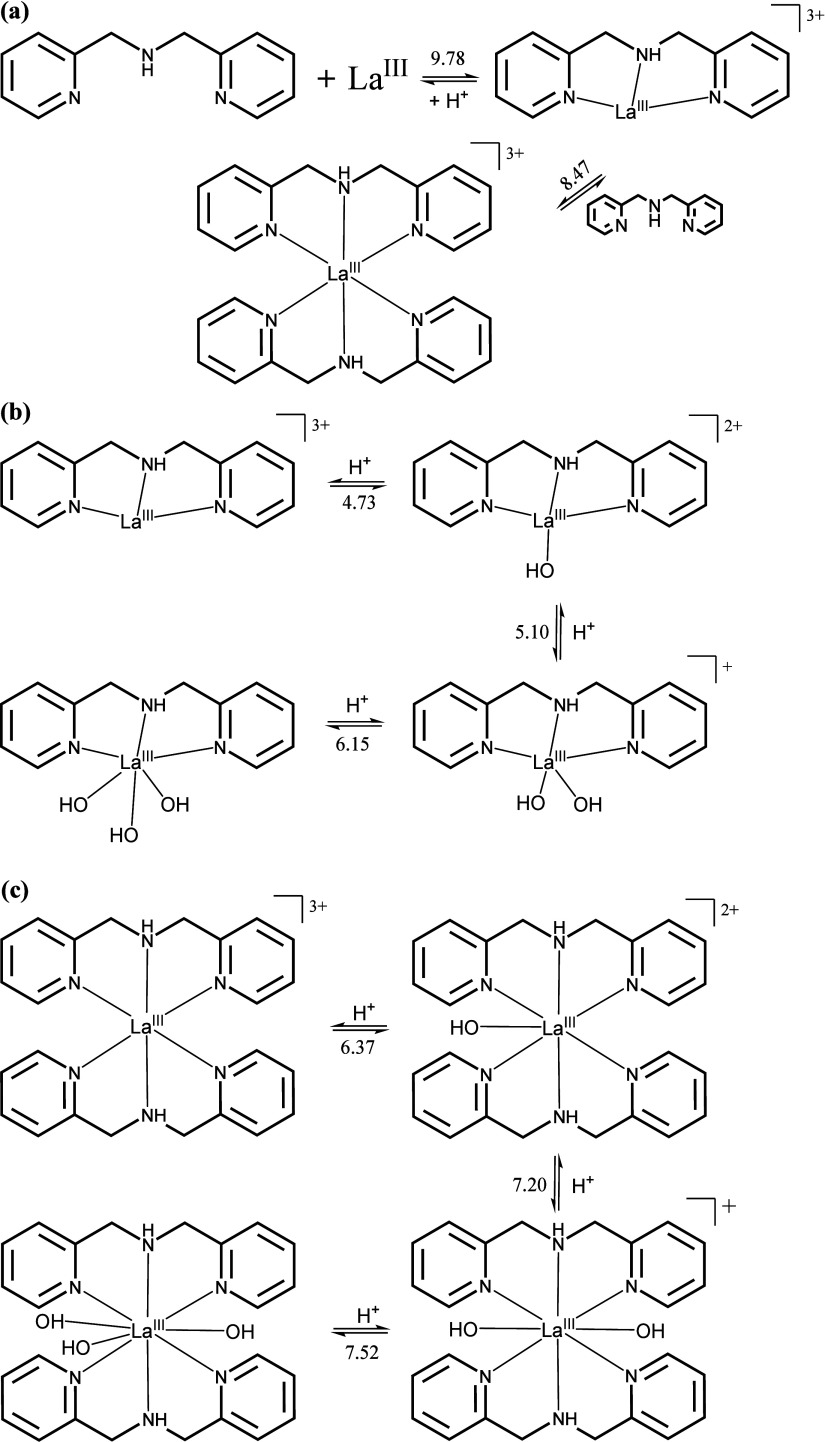
Equilibrium reactions in the complexation of L1 with La­(III), where (a) formation of the LaL1 and La­(L1)_2_ species; (b,c) hydrolysis equilibria of the LaL1 and La­(L1)_2_ complexes, leading to formation of the species: [La­(OH)­L1]^2+^, [La­(OH)_2_L1]^+^, La­(OH)_3_L1, [La­(OH)­(L1)_2_]^2+^, [La­(OH)_2_(L1)_2_]^+^, and La­(OH)_3_(L1)_2_, respectively.

**Table 1:** Thermodynamic Equilibrium Constants (log *K*) of Ligands L1, L2, and the Complexes with La­(III) at 25 °C and an Ionic Strength of 0.100 M (KCl)

species	L1 and La^III^L1	L2 and La^III^L2
[HL]/[L][H^+^]	6.60 (13)^a^	6.93 (08)^a^
[H_2_L]/[HL][H^+^]	1.48 (06)	1.42 (05)
[LaL^3+^]/[La][L]	9.0 (4)	4.37 (12)
[LaL^3+^]/[La(OH)L^2+^][H^+^]	4.68 (06)	7.25 (01)
[La(OH)L^2+^]/[La(OH)_2_L^+^][H^+^]	5.32 (22)	7.39 (01)
[La(OH)_2_L^+^] /[La(OH)_3_L][H^+^]	6.25 (16)	7.89 (06)
[La(L)_2_^3+^]/[LaL][L]	7.9 (4)	
[La(L)_2_^3+^]/[La(OH)(L)_2_^2+^][H^+^]	6.36 (16)	
[La(OH)(L)_2_^2+^]/[La(OH)_2_(L)_2_^+^][H^+^]	6.62 (06)	
[La(OH)_2_(L)_2_^+^]/[La(OH)_3_(L)_2_][H^+^]	7.69 (08)	

a The numbers in parentheses represent the decimal variation of the average of the measurements.

The formation of the LaL1 and La­(L1)_2_ species is accompanied by a decrease in the p*K*_a_ of the amine group, which can be attributed to the establishment of coordination bonds between La­(III) and the ligand. This observation supports the conclusion that the interactions between the metal center and the amine groups are covalent.

The hydroxyl species appear in Figure 5b,c. The values below or next to the equilibrium arrows are the pKas of the water molecules bonded to the metal ion, for both the [LaL1]^3+^ and [La­(L1)_2_]^3+^ species. The hydroxyl species are [La­(OH)­L1]^2+^, [La­(OH)_2_L1]^+^, La­(OH)_3_L1, [La­(OH)­(L1)_2_]^2+^, [La­(OH)_2_(L1)_2_]^+^, and La­(OH)_3_(L1)_2_.

For the equilibria involving a ratio La:L 1:1, the ligand L1 exhibited higher formation constants with lanthanum­(III) ion compared to L2 (Table 1). The formation constant value observed for the formation to LaL1 is about 4.0 × 10^4^ higher than the observed for LaL2 (9.00 vs 4.37). The results can be explained by noting that L2 is a softer ligand compared to L1, primarily because of the thiophene group present in L2.

The formation of the species 1:2 La:L was observed only in the reaction containing L1. In the case of L2, the coordination of a second ligand molecule is not observed since it does not compete with hydroxide in solution. Therefore, it is proposed that L2 coordinates to La­(III) as a bidentate ligand throughout the pyridine and amine groups. On the other hand, ligand L1 behaves as a tridentate ligand (see Figure 5).

The potentiometric titration revealed the formation of three hydroxo species at the metal center site. This study supports a coordination number of the metal ion being greater than 6 as in the species [La­(OH)_2_(L1)_2_]^+^, which is expected for lanthanides.^69,70^ The hydrolysis of an additional water molecule coordinated to lanthanum in [La­(OH)_2_(L1)_2_]^+^ displaces one molecule of L1 to form the species La­(OH)_3_L1, which predominates at pH values above 8.

Analyzing the species distribution curves (Figure 6a,b) of the lanthanum complexes, it is noted that for L1, the predominant species in the acidic region is [La­(L1)_2_]^3+^, with a maximum at pH 4.47, reaching 90.4%. In the alkaline region, the predominant species is La­(OH)_3_L1 (96.5%). In the complexation of L2 with the La­(III) ion, the major species are the hydroxo: [La­(OH)_2_L2]^+^, with a maximum at pH 7.82 (37.2%) and La­(OH)_3_L2, which predominates at pH values above pH 7.8 (Figure 6b).

**Figure 6:**
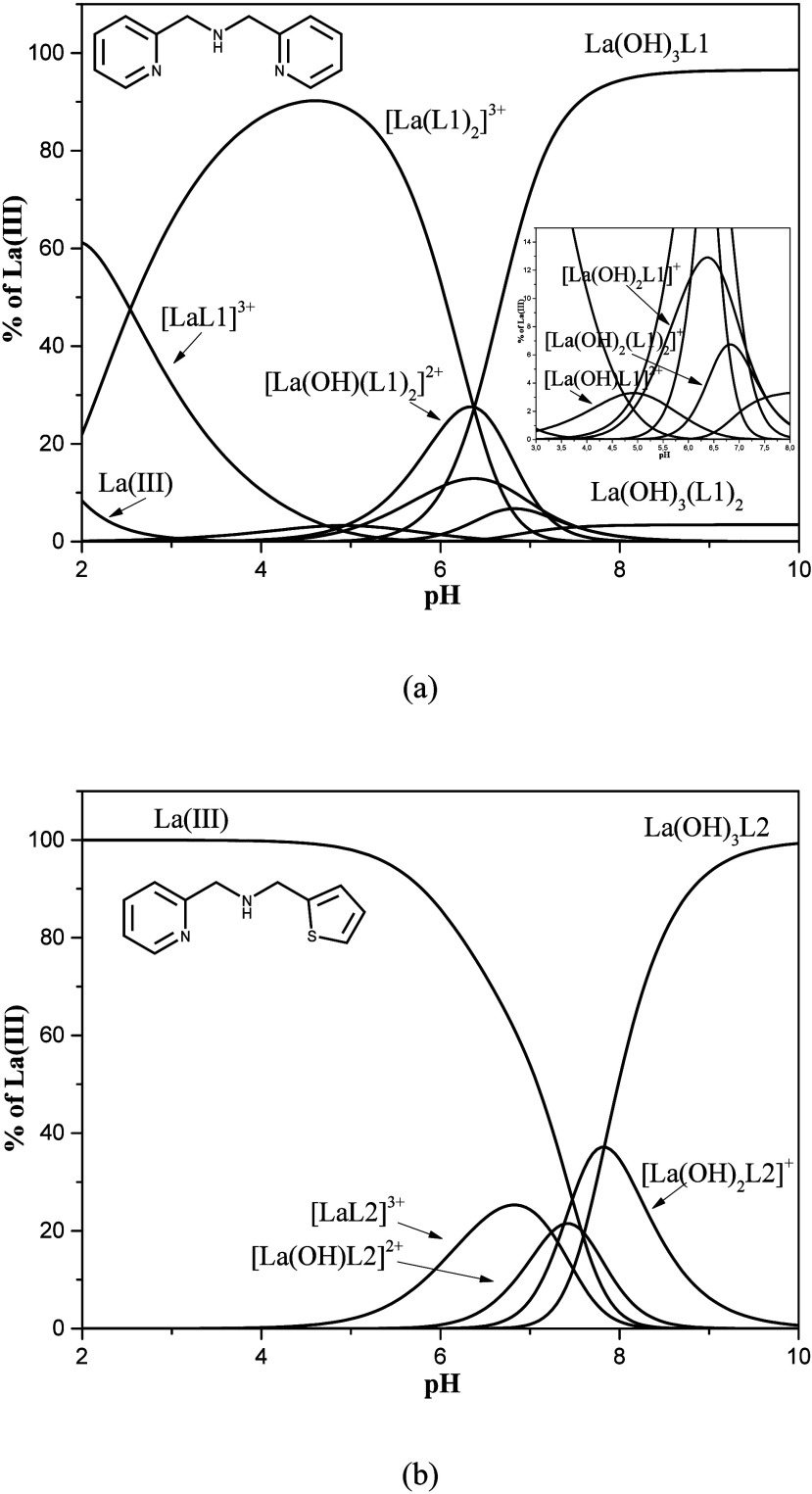
Species distribution in the complexation studies of L1 (a) and L2 (b) with La­(III).

It is important to highlight that below pH 5.0 the ligand L2 does not interact with La^3+^ ion, a behavior totally different form that shown by ligand L1, since for L1, the complex [LaL1_2_]^3+^ is the major specie in solution between pH 3 and 6. The presented data clearly show the importance of potentiometric titration studies to establish the coordination behavior of different ligands as a function of the pH.

The stronger affinity of lanthanum­(III) for ligand L1, indicated by the higher formation constants, led us to incorporate it into a solid matrix. Previously, we reported the preparation of organofunctionalized silicates with tridentate ligands, and we found that the degree of functionalization is greater in POSS systems than in traditional silicates.^45,49^ Consequently, ligand L1 was integrated into a POSS structure, resulting in the formation of a POSS-L1 compound. This aims to evaluate whether the complexation ability observed in a homogeneous system is maintained in a heterogeneous one. As presented above, the characterization data indicates that POSS composition is that shown in Figure 2 and that there are 1.5 mmol of ligand per gram of POSS.

Initially, protonation–deprotonation equilibria of the POSS-L1 were investigated. The results of the titrimetric studies of the POSS-L1 showed that its protonation constants, defined in eq 1, 9.96 (p*K*_a__1_) and 3.93 (p*K*_a__2_), were higher than the values obtained for free L1, 6.60 (p*K*_a__1_) and 1.48 (p*K*_a__2_). This is explained by changes in the ligand’s electronic structure through interactions such as hydrogen bonding with the solid matrix and the transformation of the secondary amine into a tertiary one.

Figure 7 (black line) presents the equilibrium curve of POSS-L1, showing two protonation processes involving the amine and one pyridine group.

**Figure 7:**
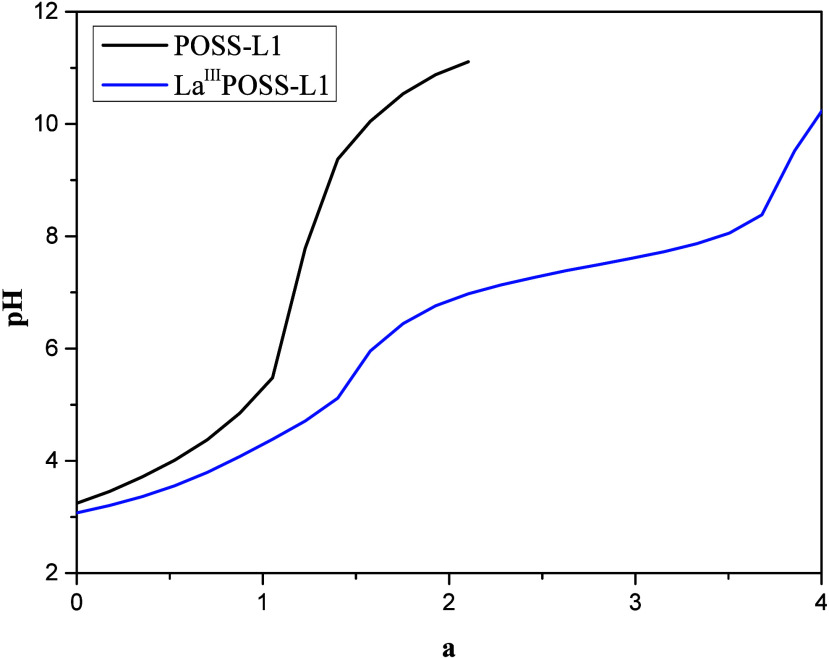
Experimental curve of the potentiometric titration of POSS-L1 and its complex with La­(III).

When analyzing the complexation results of La­(III) with the POSS-L1, it is observed that the formation constant of the 1:1 complex (Table 2), [LaPOSS-L1]^3+^ species, was slightly higher than that of the same species with free L1. Studies reported by Ramasamy and collaborators,^71^ using matrices containing amine groups, such as silica-based modified surfaces, explain interaction/adsorption mechanisms involving electrostatic forces (at more alkaline pH), ionic interactions, and coordination bonds. These interactions involved both amine groups and oxygen-containing sites on the silica surface. Therefore, oxygen-containing functionalities within the POSS framework contribute to the higher formation constant in comparison to the homogeneous system by promoting surface complexation through hydroxyl groups and enabling additional stabilization via hydrogen-bonding interactions.^72^ However, the formation of the 1:2 complex [La­(POSS-L1)_2_]^3+^ was not evidenced under conditions analogous to the homogeneous system. This is due to the greater structural rigidity in the solid system, which does not occur in the homogeneous one, which contains free L1. In the homogeneous titration system, there is a higher degree of freedom for the ligand molecules and metal ions to interact in solution. Nevertheless, the results demonstrate the efficacy of the POSS-L1 system in binding to lanthanum­(III) ion.

**Table 2:** Thermodynamic Equilibrium Constants (log *K*) of POSS-L1 and the Complexes with La­(III) at 25 °C and Ionic Strength 0.100 M (KCl)

species	POSS-L1	La^III^POSS-L1
[HL]/[L][H^+^]	9.96 (01)^a^	
[H_2_L]/[HL][H^+^]	3.93 (06)	
[LaL1^3+^]/[La][L1]		10.33 (28)^a^
[LaL1]/[La(OH)L1^2+^][H^+^]		6.45 (29)
[La(OH)L1^2+^] /[La(OH)_2_L1^+^][H^+^]		6.58 (11)
[La(OH)_2_L1^+^] /[La(OH)_3_L1][H^+^]		7.76 (06)

a The numbers in parentheses represent the decimal variation of the average of the measurements.

The titration curve of [POSS-L1] in the presence of the La­(III) ion (Figure 7, blue line) shows a decrease when compared to free POSS-L1, indicating the interaction of the metal with POSS-L1 and also the formation of hydroxyl species. All possible interactions were analyzed with the Best7 program, and the equilibrium constants determined are in Table 2. The equilibrium constants of all of the interactions detected made it possible to determine the distribution curves of all of them as a function of pH values (Figure 8). This system shows itself useful for application in the adsorption processes of this metal ion. When the pH is very acidic, there is an absence of coordinated lanthanum ions. The [LaPOSS-L1]^3+^ species are formed above pH 3, as can be seen in the species distribution diagram (Figure 8), and this indicates that lowering the pH of solution may be a strategy to promote the desorption of the La­(III).

**Figure 8:**
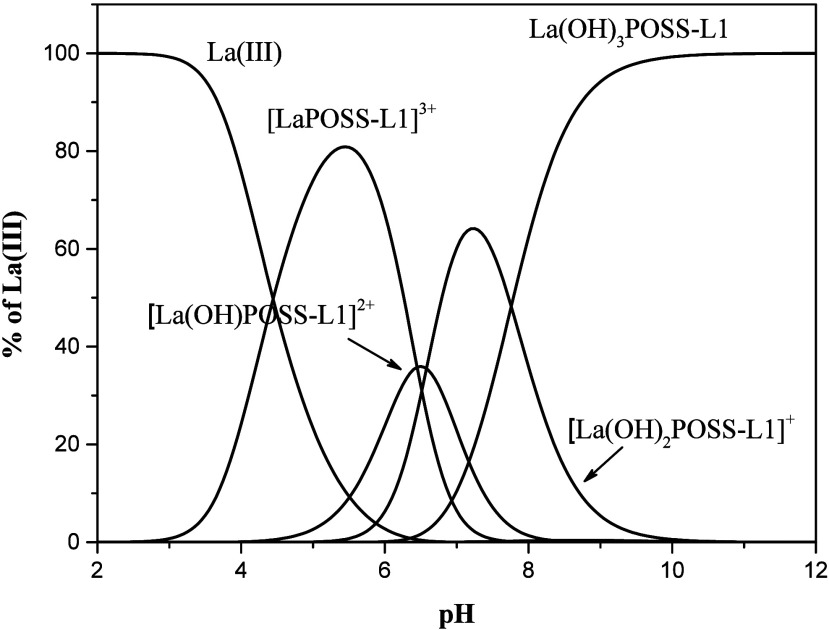
Species distribution in the complexation studies of POSS-L1 with La­(III).

The predominant species formed on the POSS-L1 surface with lanthanum­(III) are [LaPOSS-L1]^3+^ (81.0%) at pH 5.42 and La­(OH)_3_POSS-L1 above pH 8.0, demonstrating that the coordination of one molecule of ligand L1, which is anchored to the POSS, occurs with the metal in both acidic and alkaline medium. The observation that POSS-L1 maintains its coordination capability in both acidic and alkaline pH, also reported in other lanthanum­(III) complexes with ligands derived from dipicolinic acid amines,^68^ is an important finding since it may be useful to discriminate from other metal ions present in complexes matrices, since the large majority of metal ions are precipitate in neutral or alkaline pH values.^41^

## Conclusions

The potentiometric titration studies demonstrated the ability of the ligands bis­(pyridin-2-ylmethyl)­amine (L1) and (pyridin-2-ylmethyl)­(thiophen-2-ylmethyl)­amine (L2) in coordinating the lanthanum­(III) ion. The data revealed that ligand L1 is a better choice to coordinate with La­(III) ions than L2. The weaker interaction of the ligand with the thiophene group (L2) is due to the softness of this group, whereas La­(III) being a hard Lewis acid favors coordination to L1. Another important finding is that L1 forms complexes with 1:1 and 1:2 La:L1 ratios with La­(III) while L2 stabilizes just the 1:1 species.

The ability of L1 to react with La­(III) is preserved in a heterogeneous system where the POSS molecule is functionalized with L1 (referred to as POSS-L1). Notably, this heterogeneous system (POSS-L1) has a formation constant that is approximately ten times higher than that of the homogeneous system (L1). This indicates that attaching tridentate ligands to the POSS backbone is an effective strategy for capturing lanthanum-type elements. It is important to emphasize that the system POSS-L1 formed stable complexes with La­(III) at both acidic and basic pH, enabling its use in pH ranges where other metal ions (d-blocks) typically precipitate. Thus, the study of the modified POSS opens new possibilities for future research on lanthanide ion adsorption with heterogeneous systems aimed at the reuse of rare-earth elements from electronic materials.

## Supplementary Material



## Notes

^1^H NMR and Infrared spectra of the ligands and POSS-L1. The Supporting Information is available free of charge at https://pubs.acs.org/doi/10.1021/acsomega.5c06716.

(PDF)

## Abbreviations

L1: bis­(pyridin-2-ylmethyl)­amine
L2: (pyridin-2-ylmethyl)­(thiophen-2-ylmethyl)­amine
POSS-L1Matrix: polyhedral silsesquioxanes with L1
