# Supplementary material for: Attachment of Tridentate Ligand onto the Polyhedral Oligomeric Silsesquioxanes as an Efficient Strategy to Capture La(III) Ions: A Comparative Study of Homogeneous and Heterogeneous Systems Using Potentiometric Titration
Source: ACS Omega. 2025 Oct 21;10(43):51335–44. doi: 10.1021/acsomega.5c06716 (PMC12593062; doi:10.1021/acsomega.5c06716)
Supplement: Supplementary file 1 [file ao5c06716_si_001.pdf]

Supporting Information

Attachment of Tridentate ligand onto the Polyhedral Oligomeric Silsesquioxanes as an Efficient Strategy to Capture La(III) ions: a Comparative Study of Homogeneous and Heterogeneous Systems Using Potentiometric Titration

Débora de Freitas Brotto[a], Iago de Souza Reis[a], Adolfo Horn Jr[a]., Bruno Szpoganicz[a*]

[a] Department of Chemistry, Universidade Federal de Santa Catarina (UFSC), Avenue Desembargador Vitor Lima (s/n), 88040-900, Florianópolis, SC, Brazil.

*E-mail: bruno.s@ufsc.br

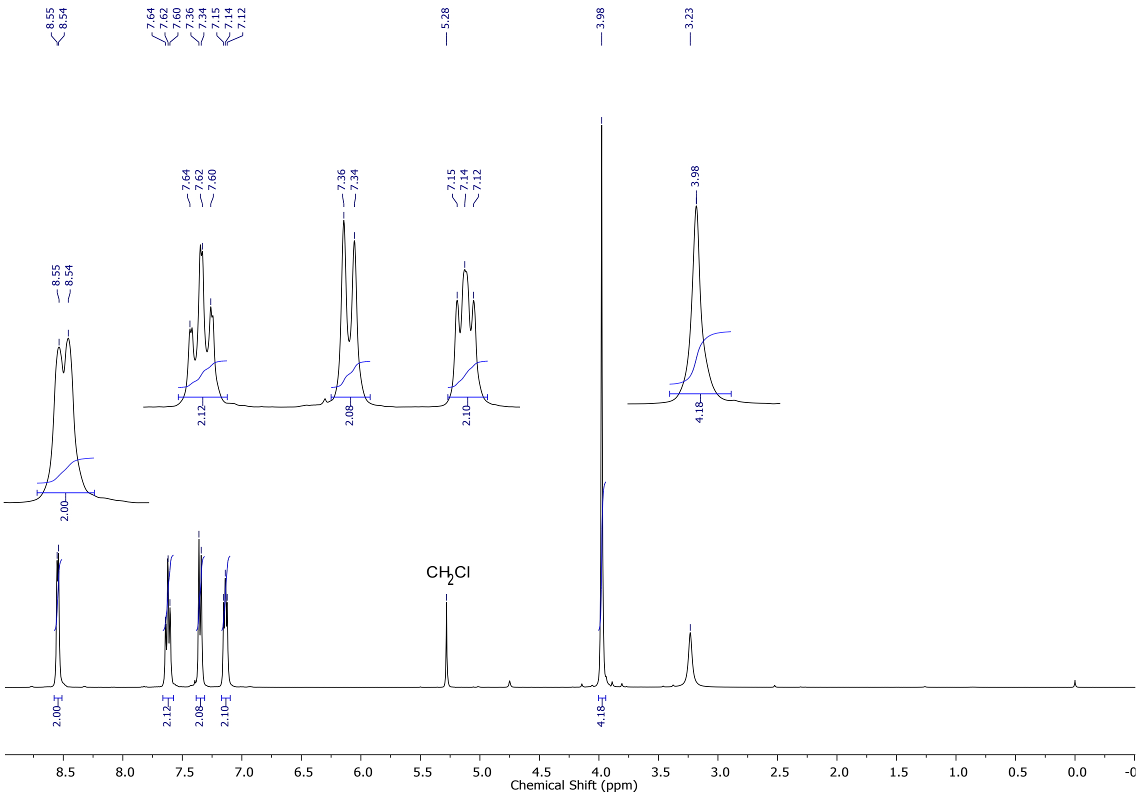

Figure S1. ^{1}H NMR spectrum of ligand 1 [bis-(pyridin-2-ylmethyl)amine].

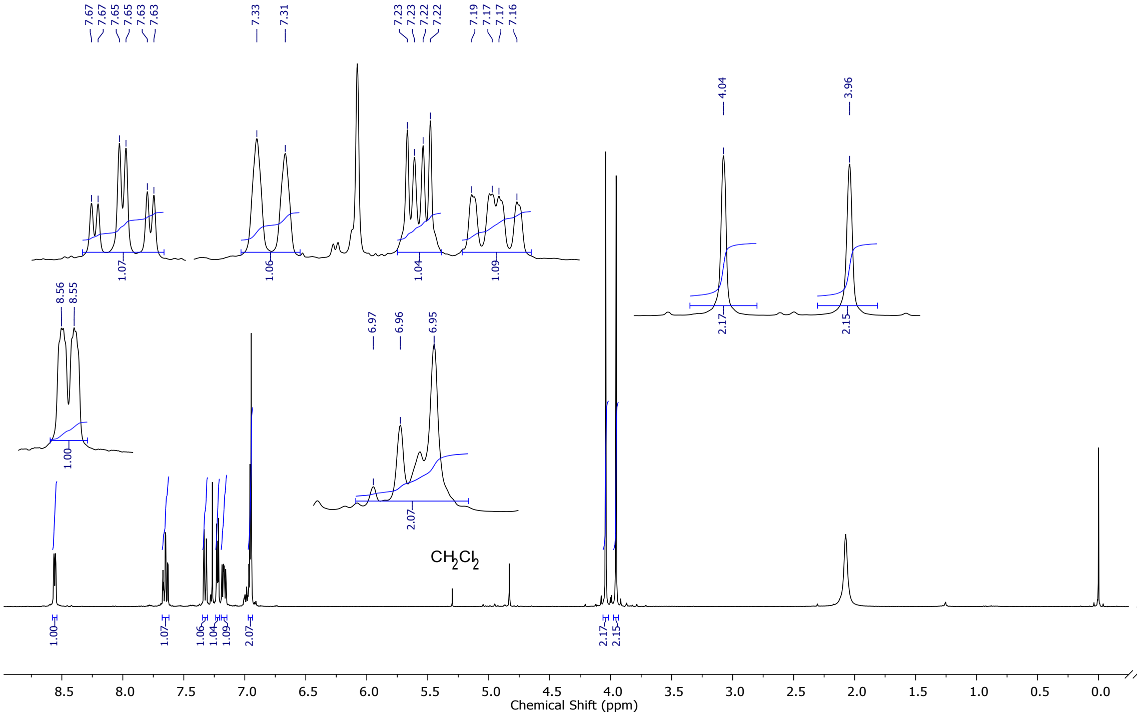

Figure S2. ^1H NMR spectrum of ligand 2 [(pyridin-2-ylmethyl)(thiophen-2-ylmethyl)amine].

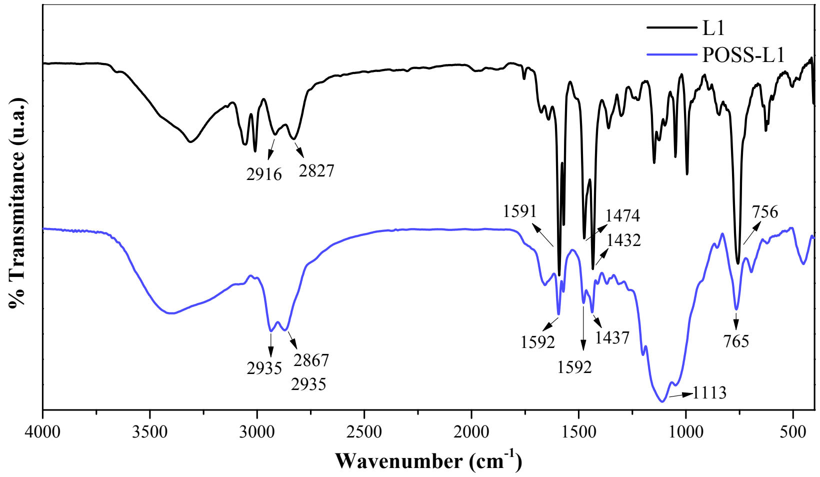

Figure S3. Comparative infrared spectrum of L1 and POSS-L1.
